# Novel Evolved Immunoglobulin (Ig)-Binding Molecules Enhance the Detection of IgM against Hepatitis C Virus

**DOI:** 10.1371/journal.pone.0018477

**Published:** 2011-04-14

**Authors:** Jie Cao, Qiuli Chen, Huaqun Zhang, Peipei Qi, Chao Liu, Xufang Yang, Niansong Wang, Baohua Qian, Jinhong Wang, Shaohua Jiang, Hua Yang, Shuhan Sun, Wei Pan

**Affiliations:** 1 Department of Microbiology, Shanghai Key Laboratory of Medical Biodefense, Second Military Medical University, Shanghai, China; 2 Department of Nephrology, Zhongshan Hospital, Fudan University, Shanghai, China; 3 Department of Nephrology, Shanghai Sixth People’s Hospital, Shanghai Jiao Tong University, Shanghai, China; 4 Department of Blood Transfusion, Changhai Hospital, Second Military Medical University, Shanghai, China; 5 Department of Cardiothoracic Surgery, The Military General Hospital of Tibet PLA, Lasa, China; 6 Shanghai Key Laboratory of Tuberculosis, Shanghai Pulmonary Hospital, Tongji University School of Medicine, Shanghai, China; 7 Department of Medical Genetics, Second Military Medical University, Shanghai, China; Saint Louis University, United States of America

## Abstract

Detection of specific antibodies against hepatitis C virus (HCV) is the most widely available test for viral diagnosis and monitoring of HCV infections. However, narrowing the serologic window of anti-HCV detection by enhancing anti-HCV IgM detection has remained to be a problem. Herein, we used LD5, a novel evolved immunoglobulin-binding molecule (NEIBM) with a high affinity for IgM, to develop a new anti-HCV enzyme-linked immunosorbent assay (ELISA) using horseradish peroxidase-labeled LD5 (HRP-LD5) as the conjugated enzyme complex. The HRP-LD5 assay showed detection efficacy that is comparable with two kinds of domestic diagnostic kits and the Abbott 3.0 kit when tested against the national reference panel. Moreover, the HRP-LD5 assay showed a higher detection rate (55.9%, 95% confidence intervals (95% CI) 0.489, 0.629) than that of a domestic diagnostic ELISA kit (Chang Zheng) (53.3%, 95% CI 0.463, 0.603) in 195 hemodialysis patient serum samples. Five serum samples that were positive using the HRP-LD5 assay and negative with the conventional anti-HCV diagnostic ELISA kits were all positive for HCV RNA, and 4 of them had detectable antibodies when tested with the established anti-HCV IgM assay. An IgM confirmation study revealed the IgM reaction nature of these five serum samples. These results demonstrate that HRP-LD5 improved anti-HCV detection by enhancing the detection of anti-HCV IgM, which may have potential value for the early diagnosis and screening of hepatitis C and other infectious diseases.

## Introduction

Serologic determination of specific antibodies against hepatitis C virus (HCV) and detection of viral RNA are the most widely available tests for viral diagnosis and monitoring of HCV infection. Since the cloning of HCV in 1989 [1], several generations of anti-HCV enzyme immunoassay (EIA) have been developed [2]–[4]. The sensitivity of the third-generation EIA was greater than 98%, a significant improvement over the approximately 80% sensitivity of the first-generation EIA, and the average seroconversion period of 10 to 16 weeks using first-generation EIA was shortened to approximately 8 weeks [3]–[7]. This improvement was mostly attributed to the addition of HCV coating antigens, core proteins (c22–3), NS3 (c33c) and NS5 to the NS4 protein (c100–3) used in the first-generation EIA.

However, this 8 weeks serologic window of anti-HCV detection remains to be a problem for early viral diagnosis and efficient screening of blood donors. It is difficult to narrow the serologic window significantly by adding new HCV coating antigens. In an individual's response against virus infection, immunoglobulin M (IgM) antibodies appear first within the first week. Therefore, it should be possible to narrow the serologic window of anti-HCV detection by enhancing the anti-HCV IgM detection.

Novel evolved immunoglobulin-binding molecules (NEIBMs), characterized by MDPL-MDPA (MDPL, mono-domain of protein L; MDPA, mono-domain of protein A), have been generated by *in vitro* molecular evolution [8], [9]. These proteins have been shown to simultaneously bind to κ light chains and V_H_3 heavy chains of immunoglobulin and have shown high affinity for IgM in addition to IgG. In this study, we developed a new anti-HCV enzyme-linked immunosorbent assay (ELISA) by using horseradish peroxidase-labeled LD5 (HRP-LD5), a NEIBM, as the conjugated enzyme complex. Comparison studies using Chinese hemodialysis samples demonstrated that HRP-LD5 based assay can improve anti-HCV detection because of its enhanced anti-HCV IgM detection efficacy.

## Materials and Methods

### Ethics statement

All aspects of the study were approved by the Shanghai Society of Medical Ethics, China and the Changhai Hospital Ethics Committee affiliated to the Second Military Medical University, China. Written informed consent was obtained from all participants in the study.

### Serum samples

A total of 195 hemodialysis patient serum samples were obtained from the hemodialysis room of Zhongshan Hospital of Fudan University (Shanghai, China; 1997). All samples were aliquoted upon receipt and stored at −20°C. The national reference panel for anti-HCV (Lot No. 0501) was from the National Institute for the Pharmaceutical and Biological Products of China and consists of 30 anti-HCV-positive human plasma samples and 30 HCV-negative serum samples that have been solvent-detergent treated. Two hundred serum specimens from healthy blood donors (Changhai Hospital, Second Military Medical University, Shanghai, China) were used to establish a cut-off value for the new diagnostic method.

### Novel evolved immunoglobulin-binding molecule

The novel evolved immunoglobulin-binding molecule (NEIBM) LD5 (B3-D-B3-D-B3) was previously generated with the characteristic structure of alternately arranged *Finegoldia magna* protein L B3 domains (B3) and staphylococcal protein A (SpA) D domains (D). LD5 showed substantially higher affinity for IgG-F(ab')2, IgM, and IgA than did either 4L (B3-B3-B3-B3) or SpA, which creates synergistic double-site binding sites to the V_H_3 and V_k_ regions of the fragment of antigen binding [8].

### Diagnostic ELISA kits and reagents

Anti-HCV diagnostic ELISA kits were purchased from Shanghai Chang Zheng Co. (China), Shanghai Ke Hua Bio-engineering Co., Ltd. (China) and Abbott Diagnostics (Abbott anti-HCV 3.0 kits, Chicago, IL, USA). Human IgG (hIgG), human IgM (hIgM), human IgA (hIgA), goat anti-human IgG, horseradish peroxidase (HRP), HRP-conjugated streptavidin, HRP-conjugated goat anti-human polyclonal polyvalent immunoglobulins (G, A, M) (HRP-goat anti-human PcAb), HRP-conjugated anti-hIgG and HRP-conjugated anti-hIgM were obtained from Sigma (St. Louis, MO, USA). hIgG, hIgM and hIgA antibodies were biotinylated using biotinyl-N-hydroxy-succinimide (Pierce, Rockford, IL, USA) in our lab (data not shown). β-mercaptoethanol (BME) was from Janssen Chimica (Geel, Belgium). Prokaryotic expressed SpA (Genbank: P02976) was kindly provided by Shanghai Fudan-Zhangjiang Bio-Pharmaceutical Co. Ltd. (Shanghai, China). HCV Nucleocapsid/NS3/NS4/NS5 recombinant proteins were from GenWay Biotech Inc. (CA, USA).

### Biosensor analyses of LD5

The binding properties of LD5 to hIgG/hIgM/hIgA were studied by surface plasmon resonance (SPR) using a Biacore 3000 instrument (Biacore, Uppsala, Sweden). Briefly, 1 mg/ml hIgG, hIgM, or hIgA resuspended in 10 mM sodium acetate (1∶5 diluted), pH 5.0, 4.5, or 4.0, was coupled to CM-5 sensor chips using amine-coupling chemistry. The association and dissociation were measured at a flow rate of 40 µl/min using NaCl/HEPES supplemented with 0.005% surfactant P20 as flow buffer. The sensor-chip surfaces were regenerated with 100 mM alanine-HCl (pH 2.5).

### HRP labeling

HRP-labeled LD5 (HRP-LD5) was prepared by using sodium periodate according to the following procedure. Five milligrams of HRP was dissolved in 1 ml distilled water, and then 200 µl of a freshly prepared solution of 0.1 M sodium periodate was added. After stirring for 20 min in the dark, the above solution was dialyzed overnight against a 1 mM sodium acetate buffer (pH 4.4) at 4°C, then 1 ml of a 5 mg/ml LD5 in a phosphate-buffered saline (PBS) (pH 7.2) was added immediately after adding a 20 µl of a 0.2 M carbonate buffer (pH 9.5). After stirring gently for 2 h in the dark, 100 µl of a 4 mg/ml sodium borohydride solution was added, and the mixture was reacted for 3 h at 4°C. At the end of the reaction, the HRP-LD5 was dialyzed overnight against 0.15 M PBS (pH 7.4) at 4°C and stored at −40°C.

### Detection of HRP-LD5 binding to Ig molecules by ELISA

hIgG, hIgM and hIgA molecules were diluted in a coating buffer (0.1 M NaHCO_3_, pH 9.6) to make a 10 µg/ml solution and then coated onto a sterile 96-well microtiter plates at 37°C for 3 h. After blocking the plates with blocking buffer (10% degreased milk powder, 0.1% Tween 20 and 0.2% mercurothiolate in 0.01 M PBS) for at least 2 h, 100 µl of 1∶2 serial dilutions of 1 mg/ml HRP-LD5 or HRP-goat anti-human PcAb were added to each well and incubated for 45 min at 37°C. Plates were developed by addition of 3, 3’, 5, 5’-tetramethylbenzidine (TMB) (Sigma, St. Louis, MO, USA), and read at 450 nm with an ELISA Reader (Bio Rad).

### Detection of anti-HCV antibody

For detection of anti-HCV using HRP-LD5, the immunoassay strips (Nunc, Rochester, NY, USA) were coated with 1.2 µg of each HCV Nucleocapsid/NS3/NS4/NS5 recombinant proteins diluted in carbonate buffer (pH 9.6). The strips were maintained at 37°C for 3 h and washed four times with a solution of PBS-Tween 20 (PBST) and blocked with blocking buffer as above. The strips were dried under reduced pressure for 2.5 h, and wrapped individually with aluminum foil under vacuumed conditions, which enable the precoated strips to be stored until use. Whenever a serum specimen was received and ready for the diagnostic test, a strip was removed from its wrapper and washed four times with PBST. Then 100 µl of a 10-fold dilution of the serum specimen, a blank, a positive and a negative reference serum sample were each added to a separate well. The strip was then placed in a 37°C incubator for 1 h. After washing four times with PBST, 100 µl of a 1,000-fold dilution of HRP-LD5 (1 mg/ml) was added to the strip and incubated for 45 min at 37°C. The strips were developed by addition of TMB and read at 450 nm in an ELISA Reader. The procedure for detection of anti-HCV IgG antibody was the same as above except for the addition of HRP-conjugated anti-hIgG instead of HRP-LD5. For anti-HCV IgM detection, goat anti-human IgG (1∶10) was added to the serum and the detection was performed using HRP-conjugated anti-hIgM instead of HRP-LD5. The anti-HCV detection by the three ELISA kits (Abbott 3.0 from Chicago, USA and both Ke Hua and Chang Zheng from Shanghai, China) was performed according to the manufacturers’ instructions.

### Confirmation of anti-HCV IgM

100 µl of the serum samples (diluted 1∶10 in PBS) were treated without or with β-mercaptoethanol (BME) at the concentration of 50 mM at 37°C for 30 min and then anti-HCV detection was performed as above using HRP-LD5 assay, anti-HCV IgM assay and Chang Zheng anti-HCV diagnostic ELISA kit.

### Qualitative detection of HCV RNA

Reverse-transcriptase nested polymerase chain reaction (RT-PCR) was performed for HCV RNA qualitative detection [10]. The HCV RNA was extracted from serum as described previously [11]. The first-strand cDNA of the highly conserved 5'-end noncoding region [12] was synthesized by RT with an antisense primer (5'-CCGGTCGTCCTGGCAATTCCGG-3') and amplified by PCR with the same antisense primer and another sense primer (5'-GGCGACACTCCACCATAGATCAC-3'). All oligonucleotides were obtained from Shanghai Sangon Biological Engineering Technology & Services Co., Ltd. (China). The PCR conditions for 35 thermal cycles were 1 min at 94°C, 2 min at 42°C, and 2 min at 72°C, followed by a 5 min extension at 72°C (Perkin Elmer Applied Biosystems, Foster City, CA, USA). PCR products were analyzed by electrophoresis in a 1.2% agarose gel and detected by staining with ethidium bromide.

### Statistical analyses

Statistical analysis between the detection rates by different diagnostic ELISA kits was performed by chi-square test. 95% confidence intervals (95% CI) are presented for the detection rates. A value of P<0.05 was considered statistically significant.

## Results

### NEIBM LD5 has significantly enhanced affinity for IgM

Our previous ELISA results had shown that LD5 molecules had much stronger binding to human immunoglobulins (Igs) than SpA [8]. In this study, binding properties of LD5 for human IgM, IgA and IgG were further detected by SPR (Table 1). LD5 showed high affinity for hIgG with an equilibrium dissociation constant (*K_D_*) of 0.89 nM. Moreover, the affinity of LD5 binding to hIgM, with a *K_D_* of 0.31 nM, was higher than that binding to hIgG and reached the level of the binding of SpA with the hIgG. LD5 also showed weak binding to hIgA (Table 1).

**Table 1:** Kinetic constants of the LD5 and SpA binding to three types of human Igs^1^.

Analyte	Constant	Ligand		
		hIgG	hIgM	hIgA
LD5	*k_a_*(M^−1^s^−1^)	(1.22±0.06)×10^5^	(7.23±0.07)×10^4^	(3.24±0.04) ×10^4^
	*k_d_*(s^−1^)	(1.08±0.05)×10^−4^	(2.21±0.05)×10^−5^	(9.44±0.11) ×10^−5^
	*K_D_*(nM)	0.89±0.003	0.31±0.01	2.91±0.07
SpA	*k_a_*(M^−1^s^−1^)	(1.54±0.12)×10^5^	(1.15±0.13)×10^5^	(4.16±0.23) ×10^5^
	*k_d_*(s^−1^)	(4.40±0.31)×10^−5^	(8.17±0.13)×10^−4^	(2.92±0.08) ×10^−3^
	*K_D_*(nM)	0.29±0.01	7.15±0.67	7.03±0.22

1 Binding experiments were performed by surface plasmon resonance with serial 1∶2 dilutions of LD5 and SpA from 1.25 and 0.8 µM for binding to hIgA, hIgG and hIgM. *k_a_*, association rate constants; *k_d_*, dissociation rate constants; *K_D_*, equilibrium dissociation constants. Data shown are averages ± standard deviation of three independent experiments.

### Binding properties of HRP-LD5 to human Igs

**Figure 1:**
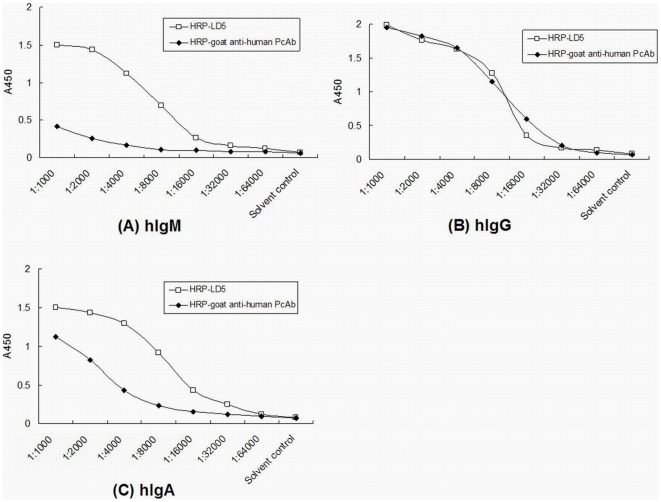
The enzyme-labeled LD5 exhibits enhanced binding activities for IgM and IgA. The binding activities of horseradish peroxidase (HRP)-labeled LD5 (HRP-LD5) or HRP-conjugated goat anti-human polyclonal antibodies (HRP-goat anti-human PcAb) to coated hIgM (A), hIgG (B) or hIgA (C) were examined by ELISA. The coating buffer was used as solvent control.

The LD5 conjugate HRP-LD5 was produced to compare its human Igs binding properties with the binding of HRP-goat anti-human PcAb, which is routinely used in most commercial anti-HCV diagnostic ELISA kits. ELISA results showed that HRP-LD5 had a much stronger binding reaction with hIgM and hIgA than did HRP-goat anti-human PcAb, and had similar binding to hIgG compared with HRP-goat anti-human PcAb (Fig. 1).

### HRP-LD5 based EIA enhance the detection of anti-HCV IgM

An anti-HCV ELISA assay was established using HRP-LD5 as conjugate and 200 serum specimens from healthy blood donors were tested to establish a cut-off value for this assay. The A_450_ values ranged from 0.017 to 0.093 (0.062±0.023). Therefore, 0.200, the average plus 6-fold of the standard deviation value, was chosen to distinguish positive from negative tests. This assay was compared with two kinds of domestic diagnostic kits (Chang Zheng and Ke Hua) and the Abbott 3.0 kit against the national reference panel (Lot No. 0501) (Table 2). All 30 HCV-positive reference serum samples were detected as positive with the HRP-LD5 assay and with the Abbott 3.0 kit, whereas one was not positive, according to the two domestic kit assays. All 30 HCV-negative reference serum samples were negative in the domestic kit assays, whereas one was detected as positive in Abbott 3.0 kit and HRP-LD5 assays (Table 2). The relative sensitivity and specificity of detection of the HRP-LD5 based assay and the domestic diagnostic kits and Abbott 3.0 kit were above 96%, which is the national standard for batch production quality control.

**Table 2:** Comparison of anti-HCV detection against the national reference panel of HRP-LD5 assay with three commercial diagnostic kits.

HRP-conjugated molecules	True positive(a)	False negative(b)	True negative(c)	False positive (d)	Sensitivity(%) a/(a+b) (95% CI)^1^	Specificity(%) c/(c+d) (95% CI)^1^	Relative accuracy (%) (a+c)/(a+b+c+d)
**Abbott 3.0 kit**	30	0	29	1	100 (0.88–1.00)	96.66 (0.83–1.00)	98.33
**Ke Hua kit**	29	1	30	0	96.66 (0.83–1.00)	100 (0.88–1.00)	98.33
**Chang Zheng kit**	29	1	30	0	96.66 (0.83–1.00)	100 (0.88–1.00)	98.33
**HRP-LD5 assay**	30	0	29	1	100 (0.88–1.00)	96.66 (0.83–1.00)	98.33

1 95% CI, 95% confidence interval.

**Figure 2:**
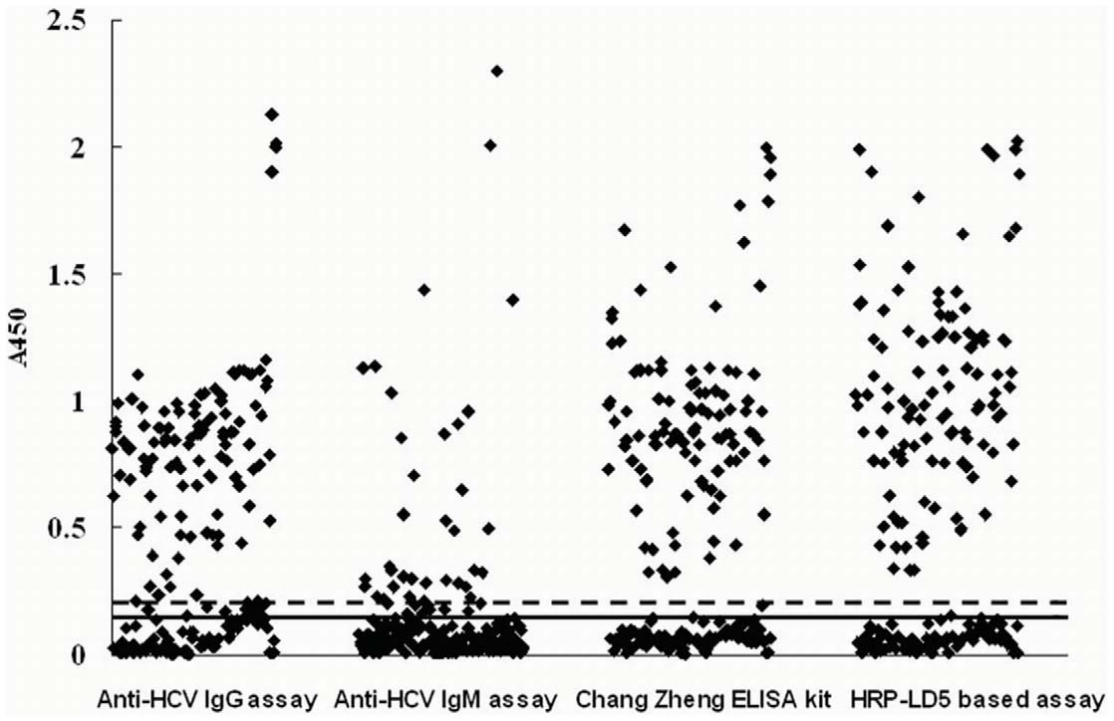
Anti-HCV detection in 195 serum specimens from hemodialysis patients by different ELISA assays. 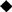
, A_450_ values, with ELISA cut-off values of either 0.150 indicated in solid line (established anti-HCV IgG assay and Chang Zheng anti-HCV diagnostic ELISA kit) or 0.200 indicated in dotted line (established anti-HCV IgM assay and HRP-LD5 based assay).

To test the detection efficacy of the HRP-LD5 based assay, we did a comparison study in an HCV high-risk population, hemodialysis patients. We also established an anti-HCV IgG assay and an anti-HCV IgM assay as reference tests. The cut-off value of the anti-HCV IgG and anti-HCV IgM ELISA were determined as above, and were 0.015 and 0.200 respectively. Serum samples from 195 hemodialysis patients were measured using the HRP-LD5 based assay, anti-HCV diagnostic ELISA kit (Chang Zheng), the established anti-HCV IgG assay and anti-HCV IgM assay (Fig. 2, Table 3). The results showed that the HRP-LD5 assay had a statistically higher positive detection rate (55.9% (109/195), 95% CI 0.489, 0.629) than that of a domestic diagnostic ELISA kit (Chang Zheng) (53.3% (104/195), 95% CI 0.463, 0.603) (P-value<0.05), and 108 were positive according to the anti-HCV IgG assay or the anti-HCV IgM assay. Interestingly, 104 samples that were positive using the anti-HCV IgG assay were scored as being positive by both the HRP-LD5-based assay and the Chang Zheng kit, and 33 were positive based on the anti-HCV IgM assay. The five serum samples which were positive by HRP-LD5 based assay and negative by Chang Zheng kit were also not detectable by the Abbott 3.0 kit (data not shown). All of the five positive for HRP-LD5 were positive for HCV RNA by RT-PCR (data not shown). However, only four of the samples that produced a false negative by the Chang Zheng kit were positive in the established anti-HCV IgM assay. The serum samples of the five patients that were false negative by the Chang Zheng kit were collected again 3 weeks later and were positive according to the Chang Zheng kit (Table 4) and the Abbott 3.0 kit (data not shown).

To further verify that these five serum samples positive in the HRP-LD5 based assay were due to specific anti-HCV IgM, the IgM confirmation test for these samples and the samples from the same patients three weeks later were performed. The A_450_ values of all five original samples were negative when the samples were treated with BME to destroy IgM (Table 4). The A_450_ values of second set of samples dramatically decreased when measured with both the HRP-LD5-based assay and the Chang Zheng kit when the samples were treated with BME, and these decreases corresponded well to the destruction of IgM as demonstrated by IgM detection assay (Table 4). These results demonstrated that the higher anti-HCV sensitivity of the HRP-LD5 based assay was due to enhanced IgM detection.

**Table 3:** Comparison of anti-HCV antibody detection in serum samples from 195 hemodialysis patients of HRP-LD5 based assay with Chang Zheng anti-HCV kit.

The established anti-HCV IgG assay or IgM assay		Chang Zheng kit		HRP-LD5 assay	
		+	−	+	−
IgG(+) or gM(+)^1^	108(55.4%)	104(53.3%)	4 (2.1%)	108(55.4%)	0
95% CI^2^	0.484–0.624	463–0.603	0.001–0.041	0.484–0.624	
IgG(+), IgM(+)^1^	33(16.9%)	33(16.9%)	0	33(16.9%)	0
95% CI^2^	0.116–0.222	0.116–0.222		0.116–0.222	
IgG(+), IgM(−)^1^	71(36.4%)	71(36.4%)	0	71(36.4%)	0
95% CI^2^	0.296–0.432	0.296–0.432		0.296–0.432	
IgG(−), IgM(+)^1^	4(2.1%)	0	0	4(2.1%)	0
95% CI^2^	0.001–0.041			0.001–0.041	
IgG(−), IgM(−)^1^	87(44.6%)	0	87(44.6%)	1(0.5%)	86(44.1%)
95% CI^2^	0.376–0.516		0.376–0.516	−0.005–0.015	0.371–0.511
Total samples	195	104	91	109	86

1 Data shown are the number of cases with each test result. Data shown in parentheses are the percentages of detected cases in the 195 total serum samples.

2 95% CI, 95% confidence interval.

**Table 4:** IgM confirmation detection of five cases of HRP-LD5 assay positive alone serum samples*.

No. of individual serum	First collected samples						Second collected samples (after three weeks)						
	Chang Zheng kit		HRP-LD5 based assay		anti-HCV IgM assay		Chang Zheng kit			HRP-LD5 based assay		anti-HCV IgM assay	
	BME		BME		BME		BME			BME		BME	
	−	+	−	+	−	+	−	+		−	+	−	+
1	0.110	0.078	0.433	0.054	0.216	0.048	0.503		0.258	1.354	0.372	1.121	0.053
2	0.178	0.060	0.358	0.047	0.187	0.064	0.280		0.263	0.957	0.675	0.565	0.042
3	0.021	0.045	0.465	0.049	0.312	0.071	0.287		0.117	1.255	0.136	1.010	0.039
4	0.142	0.076	0.876	0.068	0.550	0.055	0.803		0.426	1.876	0.463	1.233	0.064
5	0.090	0.053	0.332	0.032	0.228	0.051	0.652		0.289	1.479	0.244	0.980	0.057

*Serum samples treated without (−) or with (+) β-mercaptoethanol (BME) were used to test anti-HCV IgM antibodies. Data shown are the A_450_ values, and the cut-off values of the ELISA were either 0.200 (HRP-LD5 based assay and established anti-HCV IgM assay) or 0.150 (Chang Zheng kit).

## Discussion

The serologic window between HCV infection and the detection of specific antibodies varies from patient to patient. With current assays, seroconversion occurs on average between 7 and 8 weeks after onset of an infection [13]–[15]. About 30% to 50% of patients have undetectable anti-HCV antibodies at the onset of clinical symptoms [16]. It is widely accepted that IgM provides a first line of defense during microbial infections and IgM detection has proven valuable for early diagnosis of many viral infections [17]. Therefore, it is predicted that improved IgM detection by an anti-HCV EIA assay may narrow this serologic window.

The IgM detection efficacy of current anti-HCV assays using monoclonal or polyclonal HRP-labeled anti-human Ig antibodies as conjugates has not been evaluated. In this study, we used a NEIBM, LD5 which showed high affinity for IgM in addition to IgG (Table 1) [8], to make conjugates, HRP-LD5. The HRP-LD5 also expressed a much stronger binding capacity to hIgM than did HRP-goat anti-human PcAb (Fig. 1). Consistently, the established HRP-LD5-based anti-HCV assay showed a higher detection rate than that of the commercial diagnostic kit (Chang Zheng) in hemodialysis patients (Table 3, Fig. 2), and it was attributed to the enhanced IgM detection (Table 3, Table 4).

Hemodialysis patients are a high-risk population for HCV infection. The anti-HCV prevalence seen in these patients varied from 3.3% in New Zealand [18], 39% in South America [19], 44%–60% in Far-Eastern countries [20] to as high as 80.0% in Egypt [21]. HCV is highly transmissible among these patients, and HCV infections at many different stages, including recent infections, are observed in this population. Therefore, a comparison study was conducted among them. Our results showed that the HRP-LD5 based anti-HCV assay had a statistical significant higher detection efficacy (55.9% positive detection rate) than did the domestic diagnostic kit (Chang Zheng) (positive detection rate of 53.3%) (Table 3).These positive detection rates are consistent with the 59.7% and 58.8% anti-HCV prevalence reported in other Chinese hemodialysis patient studies [22], [23].

The five patients who tested positive using the HRP-LD5 based assay alone were demonstrated to have anti-HCV IgM antibodies and HCV RNA. Follow-up samples taken from these patients three weeks later were positive using both the HRP-LD5-based assay and the Chang Zheng kit (Table 4). These results indicate that these five patients were likely to be newly HCV infected cases, which account for 2.56% of the 195 hemodialysis patients in this study, consistent with the result in a follow-up study of Chinese Shanghai hemodialysis patients that showed the anti-HCV seroconversion rates at 6, 12, 18, 24 and 30 months were 6.4%, 11.9%, 20.7%, 35.4% and 54.5%, respectively [22].

Indirect EIAs have been developed to detect IgM against many microbes. Low sensitivity or specificity is common problem for IgM detection, and the lack of applicable anti-hIgM monoclonal and polyclonal antibodies is a major obstacle for effective IgM detection [24], [25]. An indirect anti-HCV IgM EIA assay has also been developed and extensively studied. Many reports have shown it to be useful for early diagnosis of HCV infection [26]–[29], whereas others do not support this conclusion [30], [31]. For unknown reasons, commercially available anti-IgM antibodies have low affinity constants (Ka), varying from 2×10^5^ to 5.34×10^8^ M^−1^ [32], which is much lower than that of anti-hIgG monoclonal and polyclonal antibodies, which is usually above 1×10^9^ M^−1^ [33]–[35]. Unlike the anti-IgM antibodies used in anti-HCV IgM ELISA assay, LD5 has well defined binding mode, i.e., simultaneously bound to the κ light chain and V_H_3 of hIgM. Consequently, it showed a high binding affinity to hIgM with the affinity constant (Ka) of 3.23×10^9^ M^−1^ and an equilibrium dissociation constant (K_D_) of 0.31 nM, which was as high as that of SpA to hIgG with the Ka of 3.45×10^9^ M^−1^ and the K_D_ of 0.29 nM (Table 1). Consistent with this binding property, the HRP-LD5 based assay displayed a higher IgM detection efficacy than the established IgM assay (Table 3, Table 4).

To our knowledge, this is the first report of the application of an NEIBM, LD5, to establish an anti-HCV detection assay with enhanced detection efficacy due to improved IgM detection. Our results also imply that NEIBMs with a high affinity to IgM could have potential application for detection of antibodies against other viral infections, such as hepatitis E virus or dengue virus, to improve of detection efficacy and early diagnosis.
